# Trichloridotris{*N*-[phen­yl(pyridin-2-yl)­methyl­idene]hydroxyl­amine-κ^2^ *N*,*N*′}samarium(III)

**DOI:** 10.1107/S1600536812008100

**Published:** 2012-02-29

**Authors:** Tao Lei, Wenqian Chen, Yanmei Chen, Bin Hu, Yahong Li

**Affiliations:** a Qinghai Institute of Salt Lakes, Chinese Academy of Sciences, Xining 810008, People’s Republic of China; b Key Laboratory of Organic Synthesis of Jiangsu Province, College of Chemistry, Chemical Engineering and Materials Science, Soochow University, Suzhou, 215123, People’s Republic of China

## Abstract

The Sm^III^ ion in the title compound, [SmCl_3_(C_12_H_10_N_2_O)_3_], shows a coordination number of nine with a slightly distorted tricapped trigonal prismatic geometry based on a Cl_3_N_6_ donor set. The mol­ecular structure is stabilized by three intra­molecular O—H⋯Cl hydrogen bonds.

## Related literature

For related literature on the phenyl-2-pyridyl ketone oxime ligand chelating one metal centre, see: Yin & Liu (2009 ▶); Yan & Liu (2009 ▶); Xiang *et al.* (2006 ▶); Milios *et al.* (2004 ▶). For the phenyl-2-pyridyl ketone oxime ligand bridging two metals, see: Liu *et al.*. (2011 ▶); Holynska & Dehnen (2011 ▶); Papatriantafyllopoulou *et al.* (2007 ▶). For the applications of phenyl-2-pyridyl ketone oxime complexes, see: Korpi *et al.* (2005 ▶); Stamatatos *et al.* (2006 ▶).
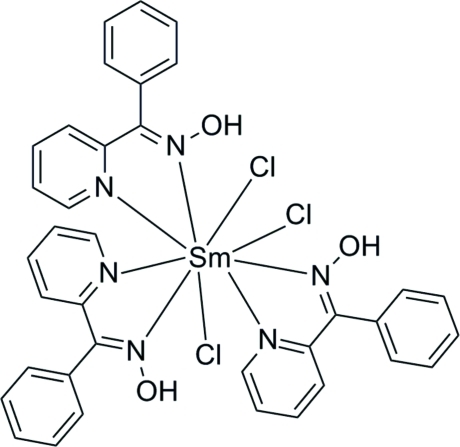


## Experimental

### 

#### Crystal data

[SmCl_3_(C_12_H_10_N_2_O)_3_]
*M* *_r_* = 851.37
Triclinic, 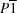

*a* = 8.6415 (17) Å
*b* = 10.422 (2) Å
*c* = 19.771 (4) Å
α = 92.18 (3)°
β = 94.47 (3)°
γ = 92.62 (3)°
*V* = 1771.8 (6) Å^3^
*Z* = 2
Mo *K*α radiation
μ = 1.93 mm^−1^
*T* = 293 K
0.30 × 0.17 × 0.12 mm

#### Data collection

Bruker SMART CCD area-detector diffractometer
Absorption correction: multi-scan (*SADABS*; Bruker, 2000 ▶) *T*_min_ = 0.595, *T*_max_ = 0.802
32032 measured reflections
8553 independent reflections
7752 reflections with *I* > 2σ(*I*)
*R*_int_ = 0.073

#### Refinement

*R*[*F* ^2^ > 2σ(*F* ^2^)] = 0.029
*wR*(*F* ^2^) = 0.068
*S* = 1.04
8553 reflections
445 parameters
H-atom parameters constrained
Δρ_max_ = 0.97 e Å^−3^
Δρ_min_ = −0.62 e Å^−3^

### 

Data collection: *SMART* (Bruker, 2000 ▶); cell refinement: *SAINT* (Bruker, 2000 ▶); data reduction: *SAINT*; program(s) used to solve structure: *SHELXS97* (Sheldrick, 2008 ▶); program(s) used to refine structure: *SHELXL97* (Sheldrick, 2008 ▶); molecular graphics: *SHELXTL* (Sheldrick, 2008 ▶); software used to prepare material for publication: *SHELXTL*.

## Supplementary Material

Crystal structure: contains datablock(s) I, global. DOI: 10.1107/S1600536812008100/tk5061sup1.cif

Structure factors: contains datablock(s) I. DOI: 10.1107/S1600536812008100/tk5061Isup2.hkl

Additional supplementary materials:  crystallographic information; 3D view; checkCIF report

## Figures and Tables

**Table 1:** Selected bond lengths (Å)

Sm1—N1	2.578 (2)
Sm1—N2	2.634 (2)
Sm1—N6	2.649 (2)
Sm1—N4	2.668 (2)
Sm1—N5	2.673 (2)
Sm1—N3	2.713 (2)
Sm1—Cl3	2.7501 (9)
Sm1—Cl4	2.7658 (9)
Sm1—Cl2	2.8114 (10)

**Table 2:** Hydrogen-bond geometry (Å, °)

*D*—H⋯*A*	*D*—H	H⋯*A*	*D*⋯*A*	*D*—H⋯*A*
O1—H1⋯Cl3	0.82	2.22	2.960 (2)	150
O2—H2⋯Cl4	0.82	2.18	2.920 (2)	150
O3—H3⋯Cl2	0.82	2.18	2.920 (2)	149

**Fig. 1.**
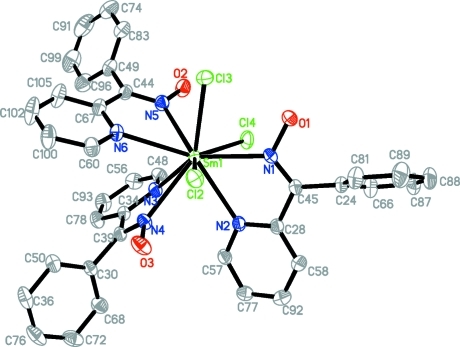
The molecular structure of the title compound, with atom labels and 30% probability displacement ellipsoids. The H atoms have been omitted for clarity.

**Fig. 2.**
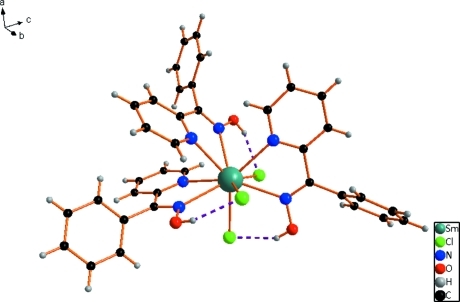
Idealised view of the molecular structure showing intramolecular hydrogen bonds as dashed lines

## supplementary crystallographic information

### Comment

Phenyl-2-pyridyl ketone oxime is a ligand used in the synthesis of metal-organic complexes. It usually binds to metals in a bidentate fashion, either chelating one metal centre (Yin & Liu, 2009; Yan & Liu, 2009; Xiang *et al.*, 2006; Milios *et al.*, 2004) or bridging two metals (Liu *et al.*, 2011; Holynska & Dehnen, 2011; Papatriantafyllopoulou *et al.*, 2007). Its complexes find applications in diverse areas such as functional supramolecular design, magnetic materials and catalysis (Korpi *et al.*, 2005; Stamatatos *et al.*, 2006). However, these complexes are focused on transition metals. Here we report a new samarium complex, which is formed by the reaction of SmCl_3_.6H_2_O with phenyl-2-pyridyl ketone oxime. The compound consists of three *N*,*N*-chelating ligands and three chloride anions. The central Sm^III^ ion adopts a distorted tricapped trigonal prism geometry (Fig. 1), which is ligated by six N atoms from three different phenyl-2-pyridyl ketone oxime ligands and three Cl anions. The Sm—N and Sm—Cl bond distances are in the expected ranges of 2.578 (2)–2.713 (2) Å and 2.7501 (9)–2.8114 (10) Å, respectively (Table 1), and the bond angles around the Sm atom are in the range of 59.12 (6)–146.13 (7)°. Three intramolecular O—H···Cl hydrogen bonds are noted (see Fig. 2 and Table 2).

### Experimental

A mixture of phenyl-2-pyridyl ketone oxime (0.0200 g, 0.1 mmol), SmCl_3_.6H_2_O (0.0180 g, 0.05 mmol), and C_2_H_5_OH (2 ml) was sealed in a 6 ml Pyrex-tube. The tube was heated at 85 °C for 3 days under autogenous pressure. Cooling of the resultant solution to room temperature gave colourless crystals of the product. The crystals were collected by filtration, washed with C_2_H_5_OH (2 ml) and dried in air. Anal. Calcd for C_36_H_30_Cl_3_N_6_O_3_Sm: C, 50.79; H, 3.55; N, 9.87%. Found: C, 50.45; H, 3.25; N, 9.65%.

### Refinement

H atoms were placed in calculated positions and included in the refinement using a riding-model approximation, with C—H = 0.93 Å and O—H = 0.82 Å, and with *U*_iso_(H) = 1.2*U*_eq_(C) or 1.5*U*_eq_(O).

### Crystal data

**Table:**

[SmCl_3_(C_12_H_10_N_2_O)_3_]	*Z* = 2
*M**_r_* = 851.37	*F*(000) = 850
Triclinic, *P*1	char
Hall symbol: -P 1	*D*_x_ = 1.596 Mg m^−^^3^
*a* = 8.6415 (17) Å	Mo *K*α radiation, λ = 0.71073 Å
*b* = 10.422 (2) Å	Cell parameters from 8108 reflections
*c* = 19.771 (4) Å	θ = 2.2–28.1°
α = 92.18 (3)°	µ = 1.93 mm^−^^1^
β = 94.47 (3)°	*T* = 293 K
γ = 92.62 (3)°	Block, colourless
*V* = 1771.8 (6) Å^3^	0.30 × 0.17 × 0.12 mm

### Data collection

**Table:**

Bruker SMART CCD area-detector diffractometer	8553 independent reflections
Radiation source: fine-focus sealed tube	7752 reflections with *I* > 2σ(*I*)
Graphite monochromator	*R*_int_ = 0.073
φ and ω scans	θ_max_ = 28.0°, θ_min_ = 1.0°
Absorption correction: multi-scan (*SADABS*; Bruker, 2000)	*h* = −11→11
*T*_min_ = 0.595, *T*_max_ = 0.802	*k* = −13→13
32032 measured reflections	*l* = −26→26

### Refinement

**Table:**

Refinement on *F*^2^	Primary atom site location: structure-invariant direct methods
Least-squares matrix: full	Secondary atom site location: difference Fourier map
*R*[*F*^2^ > 2σ(*F*^2^)] = 0.029	Hydrogen site location: inferred from neighbouring sites
*wR*(*F*^2^) = 0.068	H-atom parameters constrained
*S* = 1.04	*w* = 1/[σ^2^(*F*_o_^2^) + (0.0279*P*)^2^ + 0.2554*P*] where *P* = (*F*_o_^2^ + 2*F*_c_^2^)/3
8553 reflections	(Δ/σ)_max_ = 0.002
445 parameters	Δρ_max_ = 0.97 e Å^−^^3^
0 restraints	Δρ_min_ = −0.62 e Å^−^^3^
0 constraints	

### Special details

**Table:**

Geometry. All e.s.d.'s (except the e.s.d. in the dihedral angle between two l.s. planes) are estimated using the full covariance matrix. The cell e.s.d.'s are taken into account individually in the estimation of e.s.d.'s in distances, angles and torsion angles; correlations between e.s.d.'s in cell parameters are only used when they are defined by crystal symmetry. An approximate (isotropic) treatment of cell e.s.d.'s is used for estimating e.s.d.'s involving l.s. planes.
Refinement. Refinement of *F*^2^ against ALL reflections. The weighted *R*-factor *wR* and goodness of fit *S* are based on *F*^2^, conventional *R*-factors *R* are based on *F*, with *F* set to zero for negative *F*^2^. The threshold expression of *F*^2^ > σ(*F*^2^) is used only for calculating *R*-factors(gt) *etc*. and is not relevant to the choice of reflections for refinement. *R*-factors based on *F*^2^ are statistically about twice as large as those based on *F*, and *R*- factors based on ALL data will be even larger.

### Fractional atomic coordinates and isotropic or equivalent isotropic displacement parameters (Å^2^)

**Table:**

	*x*	*y*	*z*	*U*_iso_*/*U*_eq_	
Sm1	0.834137 (13)	0.905657 (10)	0.251022 (6)	0.03257 (5)	
Cl2	0.72244 (8)	0.79917 (6)	0.36744 (4)	0.04910 (16)	
Cl3	0.51963 (7)	0.92727 (7)	0.22387 (4)	0.05149 (16)	
Cl4	0.89706 (9)	1.14430 (6)	0.20005 (4)	0.05223 (16)	
N3	1.0834 (2)	0.84667 (18)	0.18255 (11)	0.0400 (5)	
N1	0.7598 (2)	1.08550 (19)	0.33380 (11)	0.0420 (5)	
N4	1.0201 (2)	0.72192 (18)	0.29251 (11)	0.0395 (5)	
O1	0.6146 (2)	1.1365 (2)	0.32620 (12)	0.0609 (6)	
H1	0.5629	1.0988	0.2943	0.091*	
N2	1.0487 (2)	1.01068 (18)	0.34079 (11)	0.0380 (4)	
N5	0.7661 (2)	0.88995 (19)	0.11666 (11)	0.0436 (5)	
N6	0.7487 (2)	0.68142 (19)	0.18849 (11)	0.0424 (5)	
C24	0.8012 (3)	1.2479 (2)	0.42591 (13)	0.0413 (6)	
C28	1.0078 (3)	1.0932 (2)	0.38971 (13)	0.0387 (5)	
C30	1.2153 (3)	0.5636 (2)	0.28091 (13)	0.0387 (5)	
C34	1.1492 (3)	0.7327 (2)	0.19293 (13)	0.0377 (5)	
C36	1.2342 (4)	0.3413 (3)	0.30392 (17)	0.0619 (9)	
H36	1.1901	0.2581	0.3022	0.074*	
C39	1.1232 (3)	0.6735 (2)	0.25778 (13)	0.0364 (5)	
O2	0.7737 (3)	0.99726 (17)	0.07745 (10)	0.0563 (5)	
H2	0.8046	1.0603	0.1016	0.084*	
C44	0.7355 (3)	0.7852 (2)	0.08127 (13)	0.0421 (6)	
C45	0.8487 (3)	1.1408 (2)	0.38184 (13)	0.0388 (5)	
C48	1.1171 (3)	0.9060 (3)	0.12670 (14)	0.0492 (7)	
H48	1.0781	0.9866	0.1200	0.059*	
C49	0.7139 (3)	0.7787 (3)	0.00623 (14)	0.0488 (6)	
C50	1.1485 (3)	0.4409 (2)	0.27872 (15)	0.0492 (6)	
H50	1.0466	0.4249	0.2605	0.059*	
C56	1.2057 (3)	0.8558 (3)	0.07822 (15)	0.0551 (7)	
H56	1.2237	0.9005	0.0396	0.066*	
C57	1.1961 (3)	0.9757 (2)	0.34498 (14)	0.0430 (6)	
H57	1.2273	0.9214	0.3108	0.052*	
C58	1.1091 (3)	1.1342 (3)	0.44452 (14)	0.0512 (7)	
H58	1.0761	1.1885	0.4784	0.061*	
C60	0.7322 (3)	0.5757 (2)	0.22425 (16)	0.0523 (7)	
H60	0.7472	0.5842	0.2713	0.063*	
C66	0.8616 (4)	1.3709 (2)	0.42052 (15)	0.0540 (7)	
H66	0.9350	1.3880	0.3897	0.065*	
C67	0.7224 (3)	0.6673 (2)	0.12071 (14)	0.0456 (6)	
C68	1.3676 (3)	0.5872 (3)	0.30676 (15)	0.0504 (7)	
H68	1.4142	0.6694	0.3069	0.060*	
C72	1.4497 (4)	0.4863 (3)	0.33252 (16)	0.0613 (8)	
H72	1.5516	0.5014	0.3509	0.074*	
C74	0.5744 (4)	0.8370 (3)	−0.09703 (18)	0.0715 (9)	
H74	0.4925	0.8773	−0.1194	0.086*	
C76	1.3829 (4)	0.3653 (3)	0.33123 (16)	0.0647 (9)	
H76	1.4390	0.2987	0.3491	0.078*	
C77	1.3042 (3)	1.0162 (3)	0.39746 (15)	0.0516 (7)	
H77	1.4059	0.9906	0.3978	0.062*	
C78	1.2397 (3)	0.6766 (3)	0.14662 (14)	0.0498 (6)	
H78	1.2820	0.5977	0.1549	0.060*	
C81	0.6913 (4)	1.2258 (3)	0.47195 (15)	0.0564 (7)	
H81	0.6475	1.1433	0.4754	0.068*	
C83	0.5899 (3)	0.8375 (3)	−0.02754 (15)	0.0542 (7)	
H83	0.5172	0.8774	−0.0027	0.065*	
C87	0.8122 (4)	1.4694 (3)	0.46141 (17)	0.0631 (8)	
H87	0.8518	1.5528	0.4571	0.076*	
C88	0.7077 (4)	1.4467 (3)	0.50742 (17)	0.0643 (8)	
H88	0.6778	1.5136	0.5353	0.077*	
C89	0.6464 (4)	1.3248 (4)	0.51256 (18)	0.0729 (10)	
H89	0.5736	1.3087	0.5438	0.087*	
C91	0.6808 (6)	0.7763 (4)	−0.13346 (19)	0.0888 (13)	
H91	0.6697	0.7758	−0.1806	0.107*	
C92	1.2599 (3)	1.0943 (3)	0.44881 (16)	0.0600 (8)	
H92	1.3293	1.1199	0.4857	0.072*	
C93	1.2669 (4)	0.7385 (3)	0.08813 (16)	0.0584 (8)	
H93	1.3258	0.7013	0.0559	0.070*	
C96	0.8206 (4)	0.7178 (3)	−0.03163 (17)	0.0679 (9)	
H96	0.9036	0.6780	−0.0098	0.081*	
C99	0.8025 (5)	0.7168 (4)	−0.1020 (2)	0.0835 (11)	
H99	0.8729	0.6757	−0.1276	0.100*	
C100	0.6944 (4)	0.4556 (3)	0.19527 (19)	0.0700 (9)	
H100	0.6852	0.3848	0.2222	0.084*	
C102	0.6710 (5)	0.4416 (3)	0.1271 (2)	0.0852 (12)	
H102	0.6470	0.3608	0.1063	0.102*	
C105	0.6830 (4)	0.5498 (3)	0.08822 (18)	0.0718 (9)	
H105	0.6648	0.5428	0.0412	0.086*	
O3	1.0089 (2)	0.66543 (19)	0.35400 (10)	0.0568 (5)	
H3	0.9363	0.6948	0.3729	0.085*	

### Atomic displacement parameters (Å^2^)

**Table:**

	*U*^11^	*U*^22^	*U*^33^	*U*^12^	*U*^13^	*U*^23^
Sm1	0.03595 (7)	0.02704 (7)	0.03464 (8)	0.00444 (4)	0.00201 (5)	−0.00155 (5)
Cl2	0.0536 (4)	0.0489 (3)	0.0471 (4)	0.0086 (3)	0.0146 (3)	0.0040 (3)
Cl3	0.0391 (3)	0.0587 (4)	0.0548 (4)	0.0054 (3)	−0.0051 (3)	−0.0076 (3)
Cl4	0.0744 (5)	0.0302 (3)	0.0516 (4)	−0.0016 (3)	0.0043 (3)	0.0013 (3)
N3	0.0425 (11)	0.0322 (10)	0.0460 (13)	0.0034 (8)	0.0052 (9)	0.0048 (9)
N1	0.0362 (10)	0.0395 (11)	0.0506 (13)	0.0126 (9)	0.0015 (9)	−0.0053 (10)
N4	0.0457 (11)	0.0351 (10)	0.0391 (12)	0.0082 (9)	0.0066 (9)	0.0065 (9)
O1	0.0432 (11)	0.0652 (13)	0.0720 (15)	0.0236 (9)	−0.0080 (9)	−0.0256 (11)
N2	0.0391 (11)	0.0318 (10)	0.0430 (12)	0.0037 (8)	0.0037 (9)	−0.0021 (9)
N5	0.0520 (12)	0.0377 (11)	0.0409 (12)	0.0028 (9)	0.0027 (10)	0.0020 (9)
N6	0.0485 (12)	0.0326 (10)	0.0461 (13)	−0.0012 (9)	0.0073 (10)	−0.0015 (9)
C24	0.0470 (14)	0.0371 (12)	0.0395 (14)	0.0075 (10)	0.0027 (11)	−0.0068 (11)
C28	0.0429 (13)	0.0330 (12)	0.0404 (14)	0.0036 (10)	0.0035 (11)	0.0002 (10)
C30	0.0434 (13)	0.0342 (12)	0.0401 (14)	0.0112 (10)	0.0086 (11)	0.0028 (10)
C34	0.0402 (12)	0.0327 (11)	0.0403 (14)	0.0009 (9)	0.0038 (10)	−0.0003 (10)
C36	0.089 (2)	0.0353 (14)	0.066 (2)	0.0183 (14)	0.0260 (18)	0.0078 (14)
C39	0.0411 (12)	0.0286 (11)	0.0395 (13)	0.0043 (9)	0.0031 (10)	0.0004 (10)
O2	0.0848 (14)	0.0400 (10)	0.0428 (11)	−0.0019 (10)	−0.0029 (10)	0.0058 (8)
C44	0.0428 (13)	0.0418 (13)	0.0410 (14)	0.0001 (10)	0.0033 (11)	−0.0061 (11)
C45	0.0425 (13)	0.0333 (12)	0.0408 (14)	0.0063 (10)	0.0043 (11)	−0.0031 (10)
C48	0.0502 (15)	0.0446 (14)	0.0546 (17)	0.0031 (11)	0.0083 (13)	0.0147 (13)
C49	0.0559 (16)	0.0494 (15)	0.0398 (15)	−0.0103 (12)	0.0076 (12)	−0.0076 (12)
C50	0.0520 (15)	0.0390 (13)	0.0576 (18)	0.0066 (11)	0.0086 (13)	0.0029 (12)
C56	0.0549 (16)	0.0630 (18)	0.0492 (17)	−0.0003 (14)	0.0125 (14)	0.0134 (14)
C57	0.0387 (13)	0.0377 (12)	0.0529 (16)	0.0060 (10)	0.0048 (11)	−0.0016 (11)
C58	0.0549 (16)	0.0541 (16)	0.0433 (16)	0.0101 (13)	−0.0042 (13)	−0.0101 (13)
C60	0.0598 (17)	0.0394 (14)	0.0585 (18)	−0.0021 (12)	0.0103 (14)	0.0059 (13)
C66	0.0680 (18)	0.0403 (14)	0.0548 (18)	0.0008 (13)	0.0175 (14)	−0.0077 (13)
C67	0.0478 (14)	0.0401 (13)	0.0481 (16)	−0.0031 (11)	0.0060 (12)	−0.0061 (12)
C68	0.0437 (14)	0.0497 (15)	0.0589 (18)	0.0062 (11)	0.0071 (13)	0.0067 (13)
C72	0.0476 (16)	0.082 (2)	0.0579 (19)	0.0258 (15)	0.0084 (14)	0.0096 (16)
C74	0.083 (2)	0.074 (2)	0.054 (2)	−0.0226 (18)	−0.0092 (18)	0.0093 (17)
C76	0.079 (2)	0.0628 (19)	0.060 (2)	0.0431 (17)	0.0238 (17)	0.0182 (16)
C77	0.0392 (14)	0.0551 (16)	0.0597 (19)	0.0068 (12)	−0.0026 (13)	−0.0007 (14)
C78	0.0563 (16)	0.0450 (14)	0.0503 (16)	0.0104 (12)	0.0152 (13)	0.0003 (12)
C81	0.0697 (19)	0.0482 (15)	0.0518 (18)	−0.0029 (14)	0.0162 (15)	−0.0075 (13)
C83	0.0594 (17)	0.0545 (16)	0.0469 (17)	−0.0102 (13)	0.0011 (14)	−0.0011 (13)
C87	0.088 (2)	0.0374 (14)	0.064 (2)	0.0077 (15)	0.0093 (17)	−0.0080 (14)
C88	0.081 (2)	0.0558 (18)	0.057 (2)	0.0181 (16)	0.0106 (17)	−0.0194 (15)
C89	0.081 (2)	0.079 (2)	0.061 (2)	0.0025 (18)	0.0315 (18)	−0.0168 (18)
C91	0.123 (4)	0.098 (3)	0.041 (2)	−0.040 (3)	0.008 (2)	−0.004 (2)
C92	0.0546 (17)	0.0670 (19)	0.0555 (19)	0.0048 (14)	−0.0121 (14)	−0.0055 (15)
C93	0.0653 (19)	0.0602 (18)	0.0526 (18)	0.0054 (14)	0.0235 (15)	−0.0003 (15)
C96	0.064 (2)	0.080 (2)	0.059 (2)	−0.0016 (17)	0.0114 (16)	−0.0135 (17)
C99	0.096 (3)	0.094 (3)	0.061 (2)	−0.014 (2)	0.032 (2)	−0.024 (2)
C100	0.093 (2)	0.0343 (14)	0.082 (3)	−0.0109 (15)	0.018 (2)	0.0003 (16)
C102	0.130 (3)	0.0441 (18)	0.080 (3)	−0.0266 (19)	0.022 (2)	−0.0168 (18)
C105	0.102 (3)	0.0493 (17)	0.061 (2)	−0.0202 (17)	0.0121 (19)	−0.0149 (16)
O3	0.0663 (13)	0.0632 (12)	0.0468 (11)	0.0267 (10)	0.0195 (10)	0.0191 (10)

### Geometric parameters (Å, º)

**Table:**

Sm1—N1	2.578 (2)	C56—H56	0.9300
Sm1—N2	2.634 (2)	C57—C77	1.381 (4)
Sm1—N6	2.649 (2)	C57—H57	0.9300
Sm1—N4	2.668 (2)	C58—C92	1.384 (4)
Sm1—N5	2.673 (2)	C58—H58	0.9300
Sm1—N3	2.713 (2)	C60—C100	1.371 (4)
Sm1—Cl3	2.7501 (9)	C60—H60	0.9300
Sm1—Cl4	2.7658 (9)	C66—C87	1.386 (4)
Sm1—Cl2	2.8114 (10)	C66—H66	0.9300
N3—C48	1.331 (3)	C67—C105	1.378 (4)
N3—C34	1.356 (3)	C68—C72	1.386 (4)
N1—C45	1.276 (3)	C68—H68	0.9300
N1—O1	1.385 (2)	C72—C76	1.361 (5)
N4—C39	1.276 (3)	C72—H72	0.9300
N4—O3	1.379 (3)	C74—C91	1.373 (5)
O1—H1	0.8200	C74—C83	1.370 (4)
N2—C57	1.338 (3)	C74—H74	0.9300
N2—C28	1.348 (3)	C76—H76	0.9300
N5—C44	1.280 (3)	C77—C92	1.366 (4)
N5—O2	1.387 (3)	C77—H77	0.9300
N6—C60	1.340 (3)	C78—C93	1.376 (4)
N6—C67	1.343 (3)	C78—H78	0.9300
C24—C66	1.373 (4)	C81—C89	1.372 (4)
C24—C81	1.384 (4)	C81—H81	0.9300
C24—C45	1.482 (3)	C83—H83	0.9300
C28—C58	1.380 (4)	C87—C88	1.350 (4)
C28—C45	1.482 (3)	C87—H87	0.9300
C30—C50	1.377 (4)	C88—C89	1.365 (5)
C30—C68	1.381 (4)	C88—H88	0.9300
C30—C39	1.490 (3)	C89—H89	0.9300
C34—C78	1.380 (3)	C91—C99	1.366 (6)
C34—C39	1.472 (3)	C91—H91	0.9300
C36—C76	1.363 (5)	C92—H92	0.9300
C36—C50	1.388 (4)	C93—H93	0.9300
C36—H36	0.9300	C96—C99	1.388 (5)
O2—H2	0.8200	C96—H96	0.9300
C44—C49	1.479 (4)	C99—H99	0.9300
C44—C67	1.484 (4)	C100—C102	1.349 (5)
C48—C56	1.376 (4)	C100—H100	0.9300
C48—H48	0.9300	C102—C105	1.393 (5)
C49—C96	1.392 (4)	C102—H102	0.9300
C49—C83	1.397 (4)	C105—H105	0.9300
C50—H50	0.9300	O3—H3	0.8200
C56—C93	1.369 (4)		
			
N1—Sm1—N2	60.96 (6)	C96—C49—C83	119.2 (3)
N1—Sm1—N6	146.13 (7)	C96—C49—C44	120.2 (3)
N2—Sm1—N6	140.66 (6)	C83—C49—C44	120.5 (2)
N1—Sm1—N4	121.34 (7)	C30—C50—C36	119.5 (3)
N2—Sm1—N4	71.97 (7)	C30—C50—H50	120.2
N6—Sm1—N4	68.71 (7)	C36—C50—H50	120.2
N1—Sm1—N5	126.41 (7)	C93—C56—C48	118.5 (3)
N2—Sm1—N5	139.50 (7)	C93—C56—H56	120.7
N6—Sm1—N5	59.63 (7)	C48—C56—H56	120.7
N4—Sm1—N5	111.97 (7)	N2—C57—C77	123.4 (2)
N1—Sm1—N3	137.56 (7)	N2—C57—H57	118.3
N2—Sm1—N3	83.11 (7)	C77—C57—H57	118.3
N6—Sm1—N3	76.24 (7)	C28—C58—C92	119.6 (3)
N4—Sm1—N3	59.12 (6)	C28—C58—H58	120.2
N5—Sm1—N3	67.77 (7)	C92—C58—H58	120.2
N1—Sm1—Cl3	74.68 (6)	N6—C60—C100	123.6 (3)
N2—Sm1—Cl3	135.50 (5)	N6—C60—H60	118.2
N6—Sm1—Cl3	77.87 (5)	C100—C60—H60	118.2
N4—Sm1—Cl3	136.25 (5)	C24—C66—C87	119.5 (3)
N5—Sm1—Cl3	70.91 (6)	C24—C66—H66	120.3
N3—Sm1—Cl3	138.19 (5)	C87—C66—H66	120.3
N1—Sm1—Cl4	69.59 (5)	N6—C67—C105	122.4 (3)
N2—Sm1—Cl4	76.92 (5)	N6—C67—C44	117.1 (2)
N6—Sm1—Cl4	130.98 (5)	C105—C67—C44	120.5 (3)
N4—Sm1—Cl4	131.71 (5)	C30—C68—C72	118.9 (3)
N5—Sm1—Cl4	71.60 (5)	C30—C68—H68	120.5
N3—Sm1—Cl4	81.51 (5)	C72—C68—H68	120.5
Cl3—Sm1—Cl4	91.54 (4)	C76—C72—C68	120.7 (3)
N1—Sm1—Cl2	69.82 (5)	C76—C72—H72	119.6
N2—Sm1—Cl2	82.21 (5)	C68—C72—H72	119.6
N6—Sm1—Cl2	86.06 (5)	C91—C74—C83	119.4 (4)
N4—Sm1—Cl2	71.06 (5)	C91—C74—H74	120.3
N5—Sm1—Cl2	138.12 (5)	C83—C74—H74	120.3
N3—Sm1—Cl2	130.19 (5)	C72—C76—C36	120.4 (3)
Cl3—Sm1—Cl2	79.46 (3)	C72—C76—H76	119.8
Cl4—Sm1—Cl2	139.37 (3)	C36—C76—H76	119.8
C48—N3—C34	116.5 (2)	C92—C77—C57	119.2 (3)
C48—N3—Sm1	121.95 (16)	C92—C77—H77	120.4
C34—N3—Sm1	118.93 (16)	C57—C77—H77	120.4
C45—N1—O1	113.35 (19)	C93—C78—C34	119.4 (2)
C45—N1—Sm1	126.08 (15)	C93—C78—H78	120.3
O1—N1—Sm1	120.47 (15)	C34—C78—H78	120.3
C39—N4—O3	112.88 (18)	C89—C81—C24	120.4 (3)
C39—N4—Sm1	124.81 (16)	C89—C81—H81	119.8
O3—N4—Sm1	122.25 (13)	C24—C81—H81	119.8
N1—O1—H1	109.5	C74—C83—C49	120.5 (3)
C57—N2—C28	117.1 (2)	C74—C83—H83	119.8
C57—N2—Sm1	122.81 (16)	C49—C83—H83	119.8
C28—N2—Sm1	119.70 (15)	C88—C87—C66	121.4 (3)
C44—N5—O2	113.0 (2)	C88—C87—H87	119.3
C44—N5—Sm1	124.96 (17)	C66—C87—H87	119.3
O2—N5—Sm1	121.83 (14)	C87—C88—C89	119.3 (3)
C60—N6—C67	117.1 (2)	C87—C88—H88	120.3
C60—N6—Sm1	120.33 (18)	C89—C88—H88	120.3
C67—N6—Sm1	122.54 (16)	C88—C89—C81	120.5 (3)
C66—C24—C81	118.8 (2)	C88—C89—H89	119.7
C66—C24—C45	120.9 (2)	C81—C89—H89	119.7
C81—C24—C45	120.2 (2)	C99—C91—C74	121.5 (3)
N2—C28—C58	122.2 (2)	C99—C91—H91	119.2
N2—C28—C45	117.1 (2)	C74—C91—H91	119.2
C58—C28—C45	120.7 (2)	C77—C92—C58	118.3 (3)
C50—C30—C68	120.3 (2)	C77—C92—H92	120.9
C50—C30—C39	120.6 (2)	C58—C92—H92	120.9
C68—C30—C39	119.1 (2)	C56—C93—C78	118.8 (3)
N3—C34—C78	122.3 (2)	C56—C93—H93	120.6
N3—C34—C39	116.2 (2)	C78—C93—H93	120.6
C78—C34—C39	121.5 (2)	C99—C96—C49	119.7 (3)
C76—C36—C50	120.1 (3)	C99—C96—H96	120.1
C76—C36—H36	119.9	C49—C96—H96	120.1
C50—C36—H36	119.9	C91—C99—C96	119.7 (4)
N4—C39—C34	116.61 (19)	C91—C99—H99	120.2
N4—C39—C30	122.7 (2)	C96—C99—H99	120.2
C34—C39—C30	120.7 (2)	C102—C100—C60	119.0 (3)
N5—O2—H2	109.5	C102—C100—H100	120.5
N5—C44—C49	123.6 (2)	C60—C100—H100	120.5
N5—C44—C67	115.4 (2)	C100—C102—C105	119.1 (3)
C49—C44—C67	121.1 (2)	C100—C102—H102	120.4
N1—C45—C28	115.1 (2)	C105—C102—H102	120.4
N1—C45—C24	123.6 (2)	C67—C105—C102	118.8 (3)
C28—C45—C24	121.3 (2)	C67—C105—H105	120.6
N3—C48—C56	124.3 (2)	C102—C105—H105	120.6
N3—C48—H48	117.9	N4—O3—H3	109.5
C56—C48—H48	117.9		

### Hydrogen-bond geometry (Å, º)

**Table:**

*D*—H···*A*	*D*—H	H···*A*	*D*···*A*	*D*—H···*A*
O1—H1···Cl3	0.82	2.22	2.960 (2)	150
O2—H2···Cl4	0.82	2.18	2.920 (2)	150
O3—H3···Cl2	0.82	2.18	2.920 (2)	149
